# Diet Quality and Caloric Accuracy in AI-Generated Diet Plans: A Comparative Study Across Chatbots

**DOI:** 10.3390/nu17020206

**Published:** 2025-01-07

**Authors:** Hüsna Kaya Kaçar, Ömer Furkan Kaçar, Amanda Avery

**Affiliations:** 1 Division of Nutrition and Dietetics, Faculty of Health Sciences, Amasya University, Amasya 05100, Türkiye; husna.kacar@amasya.edu.tr; 2 Doctoral School of Health Sciences, Faculty or Health Sciences, University of Pécs, 7622 Pécs, Hungary; 3 Department of Biochemistry and Medical Chemistry, Medical School, University of Pécs, 7624 Pécs, Hungary; 4 Nutrition and Dietetics Department, Sabuncuoglu Serefeddin Training and Research Hospital, Amasya University, Amasya 05200, Türkiye; 5 Division of Nutrition, Food & Dietetics, School of Biosciences, University of Nottingham, Leics LE12 5RD, UK; amanda.avery@nottingham.ac.uk

**Keywords:** AI technology, caloric accuracy, chatbots, diet quality, personalised nutrition, weight-loss diets

## Abstract

**Background/Objectives:** With the rise of artificial intelligence (AI) in nutrition and healthcare, AI-driven chatbots are increasingly recognised as potential tools for generating personalised diet plans. This study aimed to evaluate the capabilities of three popular chatbots—Gemini, Microsoft Copilot, and ChatGPT 4.0—in designing weight-loss diet plans across varying caloric levels and genders. **Methods:** This comparative study assessed the diet quality of meal plans generated by the chatbots across a calorie range of 1400–1800 kcal, using identical prompts tailored to male and female profiles. The Diet Quality Index-International (DQI-I) was used to evaluate the plans across dimensions of variety, adequacy, moderation, and balance. Caloric accuracy was analysed by calculating percentage deviations from requested targets and categorising discrepancies into defined ranges. **Results:** All chatbots achieved high total DQI-I scores (DQI-I > 70), demonstrating satisfactory overall diet quality. However, balance sub-scores related to macronutrient and fatty acid distributions were consistently the lowest, showing a critical limitation in AI algorithms. ChatGPT 4.0 exhibited the highest precision in caloric adherence, while Gemini showed greater variability, with over 50% of its diet plans deviating from the target by more than 20%. **Conclusions:** AI-driven chatbots show significant promise in generating nutritionally adequate and diverse weight-loss diet plans. Nevertheless, gaps in achieving optimal macronutrient and fatty acid distributions emphasise the need for algorithmic refinement. While these tools have the potential to revolutionise personalised nutrition by offering precise and inclusive dietary solutions, they should enhance rather than replace the expertise of dietetic professionals.

## 1. Introduction

The integration of artificial intelligence (AI) into various aspects of daily life has brought significant advancements across multiple sectors, including healthcare, education, and nutrition [1,2]. As the prevalence of AI-driven applications continues to grow, there has been increasing interest in evaluating their efficacy and potential limitations [3]. AI has the potential to revolutionise healthcare, especially by improving the personalisation of care delivery systems [4]. High-quality, personalised diet plans are vital educational resources for weight management, playing a key role in improving clinical outcomes by offering guidance customised to each individual’s specific needs [5,6]. However, without human assistance, the development and implementation of personalised diet plans in real-world settings become a complex task, necessitating the integration of various clinical and cultural factors and posing significant challenges [7,8].

Individuals seeking to lose weight increasingly turn to chatbots for guidance, valuing their convenience and potential for personalised support [9,10]. AI-chatbots are advanced systems that use artificial intelligence techniques such as natural language processing and machine learning to simulate human-like interactions [11]. Unlike traditional chatbots, which follow predefined scripts, AI-chatbots are capable of understanding and generating context-aware responses, allowing for more dynamic and personalised communication [11,12].

These AI-based tools have garnered significant attention as promising resources for weight loss and lifestyle modification [13]. By simulating human conversation, chatbots provide tailored diet and exercise recommendations, motivational support, and encouragement to enhance adherence to weight management programmes [3,10]. Their accessibility, cost-effectiveness, and ability to deliver personalised advice position them as valuable tools in addressing obesity and promoting healthier lifestyles [9].

While traditional dietary planning has relied on healthcare professionals and evidence-based guidelines [14], recent technological developments have introduced chatbots capable of generating diet plans tailored to specific calorie requirements and health goals [9,15]. Despite the promising nature of these AI-powered tools, questions remain regarding the accuracy and quality of the diet lists they produce [16]. Studies have shown that chatbots can lead to significant weight loss outcomes, with some reporting a decrease of 1.3–2.4 kg over 12–15 weeks of use [17]. However, the overall quality of existing studies is low, and more rigorous research with larger sample sizes and longer follow-up periods is needed to establish the efficacy and safety of chatbots for weight loss [13,18]. Ensuring that such diet plans meet established nutritional standards is crucial, as suboptimal recommendations could potentially lead to nutrient deficiencies or imbalanced eating patterns [19,20].

The Diet Quality Index-International (DQI-I) is widely acknowledged as a robust and comprehensive tool for evaluating the nutritional quality of dietary patterns [21]. It serves as an effective framework for determining whether a given diet aligns with established dietary guidelines and supports overall health [22]. Its proven versatility and reliability have established its role as a cornerstone in both research and clinical practice for assessing dietary adequacy [21]. In the context of AI-generated diet plans, the DQI-I provides a critical, objective means of evaluating how well these digital recommendations adhere to recognised nutritional standards. Although some studies have explored the quality of diets generated by chatbots using evaluations conducted by dietitians [23,24,25,26,27,28], there remains a notable lack of research employing the DQI-I for this purpose. This gap is significant, as it limits our understanding of the potential for chatbots to produce nutritionally balanced and health-promoting meal plans. Given the growing reliance on AI-powered tools in personalised nutrition, the application of the DQI-I is essential for identifying potential limitations in these outputs, including nutrient imbalances or a failure to meet specific dietary needs. Addressing this research gap would provide valuable insights into the effectiveness of AI-generated diets and their ability to meet the standards of professionally designed plans.

This study seeks to explore the capabilities of various chatbots in generating weight loss diet plans of different calorie levels, with a focus on assessing their accuracy and nutritional quality. By comparing these AI-generated diet lists against the DQI-I, we aim to provide a systematic evaluation of how well these tools adhere to current dietary standards. The findings from this research will offer valuable insights into the potential and limitations of AI in the field of nutrition and may guide future improvements in digital health technologies. This investigation will also contribute to understanding the role AI could play in assisting healthcare professionals and empowering individuals in their weight loss processes.

## 2. Materials and Methods

This study employed a comparative design to evaluate and compare the diet quality of meal plans generated by Gemini, Microsoft Copilot, and ChatGPT 4.0 [29,30,31]. These chatbots were selected for their popularity and broad application as AI tools capable of producing personalised diet plans. To minimise the influence of previous user interactions, a new email account was created and used to log in to each chatbot, ensuring that each AI’s responses were unaffected by prior learning.

Each chatbot was tasked with generating unique diet plans within a calorie range of 1400–1800 kcal, with an increment of 100 kcal, tailored to male and female profiles to explore potential gender-based differences in diet quality. To ensure the relevance of our study design to clinical practice and maintain consistency across the AI-generated diet plans, we consulted registered dietitians with over 10 years of experience in a university hospital to identify commonly used calorie ranges for weight management diets. Based on their recommendation, we selected a caloric range of 1400–1800 kcal/day for chatbot diet plan queries. Age and sex were chosen as the sole parameters to simplify the queries, enabling an assessment of the general nutritional quality of the diets without introducing variability from individual preferences or cultural differences. Also, this caloric range is consistent with common dietary guidelines for weight loss [32,33,34]. Identical prompts were used across the three chatbots, modified only to specify the gender and caloric target, as follows: “Prepare a healthy daily meal plan for a female aged 35 with 1400 kcal, including portion sizes in grams”. One meal plan was generated per calorie and gender specification within each AI tool, resulting in a total of 30 diet plans.

The DQI-I was used to systematically assess various dimensions of diet quality within the meal plans, focusing on variety, adequacy, moderation, and balance [21]. Each component was carefully defined and scored (as outlined in Table 1). For *variety*, assessments included two categories: food groups and protein sources. Food variety was evaluated across five groups (meat/poultry/fish/egg, dairy/beans, grains, fruits, and vegetables), awarding 3 points if at least one item from each group was included, with a total possible score of 15 points. Protein variety considered six categories (meat, poultry, fish, dairy, beans, and eggs), with points assigned based on the number of different protein sources, up to a maximum of 5 points. *Adequacy* was scored against eight food groups—vegetables, fruit, grains, fibre, protein, iron, calcium, and vitamin C—each receiving between 0 and 5 points based on the percentage of the Recommended Daily Allowance (RDA) met, allowing a potential total of 40 points. *Moderation* focused on six dietary components, including total fat, saturated fat, cholesterol, sodium, and empty-calorie foods, with each component scored from 0 to 6 points based on adherence to recommended intake limits, contributing up to 30 points. *Balance* was evaluated through macronutrient and fatty acid ratios, with a maximum score of 10 points awarded for optimal balance. The DQI-I is determined by adding the five sub-scores, resulting in a total score ranging from 0 to 100. To ensure data accuracy, nutrient information including energy for each food item was verified using the USDA’s Food Data Central [35]. For adequacy, moderation, and balance, scoring was based on the United States’ RDAs and Dietary Reference Intakes (DRIs).

All nutritional data, including Diet DQI-I scores and sub-scores, for each diet plan generated by the chatbots, were systematically documented in an electronic database.

### Statistical Analysis

The mean and standard deviation (SD) of DQI-I scores were calculated for each subscale (*variety—food groups; variety—protein sources*; *adequacy; moderation; and balance*) as well as the total DQI-I score for each chatbot. A one-way analysis of variance (ANOVA) was conducted to compare the DQI-I subscale and total scores across the three chatbots. To examine gender-based differences, independent samples *t*-test were performed to compare the DQI-I scores of diet plans designed for males versus females within each subscale and overall. The latest version (30.0.0) of IBM SPSS Statistics software was employed for all statistical analyses.

**Figure 1:**
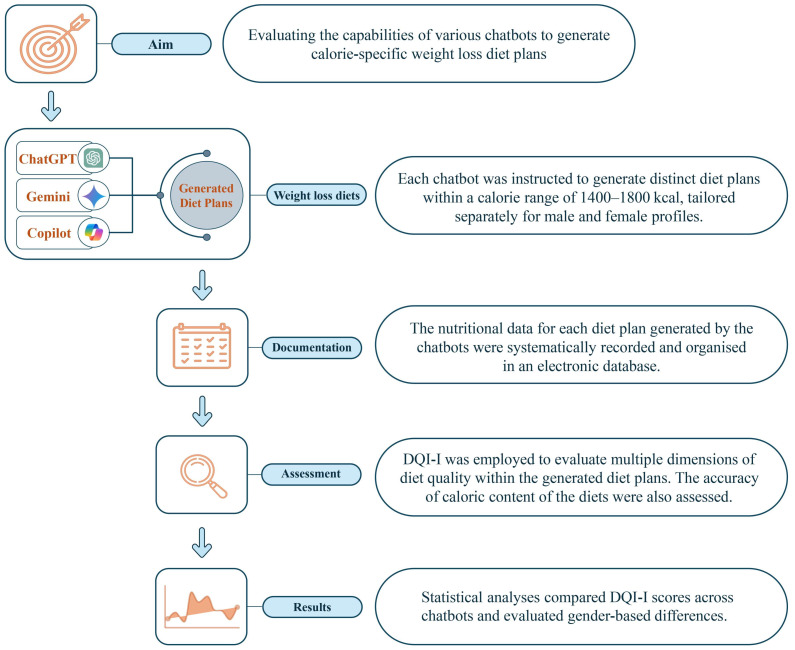
Methodological framework for assessing chatbot-generated diet plans.

Additionally, the percentage differences between the requested calorie levels and the calorie content of the diet plans generated by the chatbots were calculated and categorised into five ranges: <5%, 5–9.99%, 10–14.99%, 15–19.99%, and ≥20%. These discrepancies were reported to evaluate the precision of the chatbots in meeting caloric targets. Figure 1 illustrates the flowchart of the study methods, providing a visual representation of the key steps and processes involved in the research.

## 3. Results

**Figure 2:**
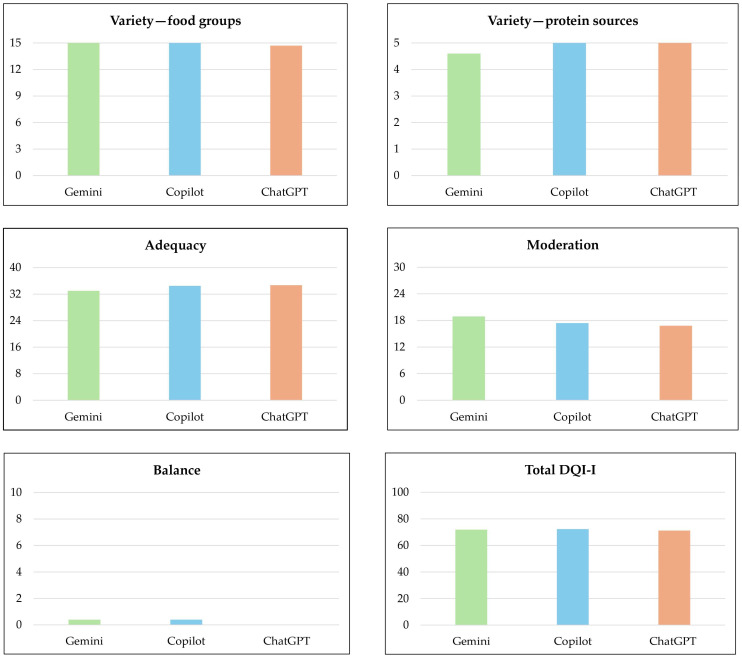
Bar charts of mean total DQI-I scores and sub-scores for Gemini, Microsoft Copilot, and ChatGPT 4.0.

The total DQI-I score for all diets generated by the chatbots (*n* = 30) was 71.80 (±4.3). The mean sub-scores were as follows: *variety*—food groups, 14.90 (±0.5); *variety*—protein sources, 4.87 (±0.5); *adequacy*, 34.07 (±2.1); *moderation*, 17.70 (±3.5); and *balance*, 0.27 (±0.9). The mean total DQI-I scores were 71.90 (±4.1) for Gemini (*n* = 10), 72.30 (±4.1) for Microsoft Copilot (*n* = 10), and 71.20 (±5.2) for ChatGPT 4.0 (*n* = 10). All diet plans generated by Gemini and Microsoft Copilot included all five food groups, achieving the maximum score for the “*variety*—food groups” subscale. For the “*variety*—protein sources” subscale, all diet plans created by Microsoft Copilot and ChatGPT 4.0 incorporated three or more protein sources, also earning the maximum score in this category. The *balance* sub-score, which evaluates the macronutrient and fatty acid ratios, was the lowest-scoring subscale across all chatbots, with mean scores of 0.40 (±4.1), 0.40 (±0.8), and 0.00 (±0.0) for Gemini, Microsoft Copilot, and ChatGPT 4.0, respectively. The one-way ANOVA revealed no statistically significant differences among the chatbots for any DQI-I sub-scores or the total DQI-I score (*p* > 0.05). The mean and SD for total DQI-I scores and sub-scores across the chatbots are presented in Table 2 and Figure 2. 

The total DQI-I scores for diet plans tailored for females (*n* = 15) and males (*n* = 15) were 71.73 (±3.9) and 71.87 (±4.9), respectively. Independent-sample t-tests indicated no statistically significant differences between the genders for total DQI-I scores. However, the mean sub-scores for “*variety*—food groups” and “*variety*—protein sources” were significantly higher for diet plans designed for females compared to those for males (*p* < 0.05) (Table 3).

**Figure 3:**
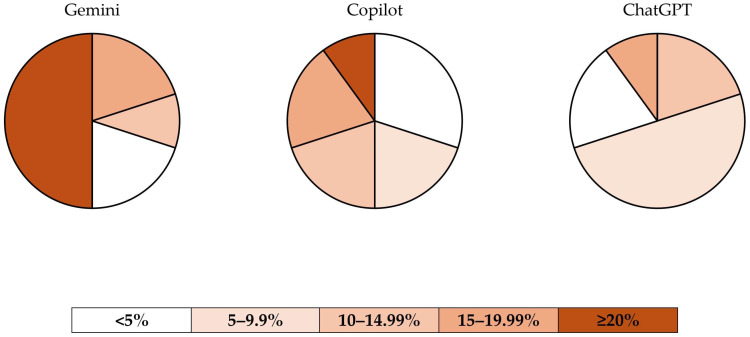
Distribution of diet plans by percentage difference between requested and generated calorie levels.

ChatGPT 4.0 demonstrated the highest precision in meeting the requested caloric targets among the three chatbots. None of the diet plans generated by ChatGPT 4.0 deviated by more than or equal to 20% from the requested calorie level. In contrast, 50% of the diet plans produced by Gemini (*n* = 5) exceeded the requested calorie target by more than or equal to 20%. Across all chatbots, the highest proportion of diet plans fell within the <5% (*n* = 7) and 5–9.99% (*n* = 7) deviation ranges. Table 4 summarises the precision of the chatbots, and Figure 3 provides pie chart visualisations of the percentage deviations.

## 4. Discussion

This study aimed to evaluate the capabilities of various chatbots in generating weight loss diet plans across different calorie levels, focusing on their accuracy in meeting caloric targets and the nutritional quality of the proposed diets, with findings highlighting their overall effectiveness and limitations as assessed using the DQI-I. Despite achieving relatively high total DQI-I scores across all chatbots, the sub-scores reveal critical areas requiring improvement. The *balance* subscale, which evaluates macronutrient and fatty acid ratios, consistently received the lowest scores. Notably, while no significant differences were observed among the chatbots for total or subscale DQI-I scores (*p* > 0.05), distinct trends in meeting specific dietary requirements emerged, with ChatGPT 4.0 demonstrating the highest precision in caloric adherence. Additionally, gender-based analysis revealed differences in the *variety* subscale scores, indicating a potential bias or variability in tailoring diets to male versus female users.

This study is the first to quantitatively assess chatbot-generated diets using the DQI-I, providing a validated and standardised measure of diet quality. Previous research has primarily relied on qualitative assessments, comparing AI-generated diets to those developed by dietitians through subjective evaluations [36]. The use of a quantitative metric such as the DQI-I in this study not only enhances the objectivity of the findings but also establishes a benchmark for evaluating the nutritional performance of AI-driven diet planning tools. However, the approach used in previous research has several limitations. Firstly, it is often subjective and can be influenced by personal biases and preferences [25,36]. Secondly, it may not capture the complexity of dietary requirements, which can lead to inaccuracies in evaluating the quality of AI-generated diets [24,26]. For instance, a study found that experts’ responses to human-designed diets were more positive when they were provided with food name information, whereas AI-generated diets were often evaluated based on nutrient information alone [36]. This highlights the need for more objective and comprehensive evaluation methods that consider both nutritional adequacy and composition style [25,28,36]. Moreover, previous studies have also been limited by their focus on single-dimensional evaluations, such as energy estimation or food classification [26,37]. However, dietary assessment is a multifaceted task that requires the consideration of various factors, including variety, adequacy, and balance [21]. By employing the DQI-I, this study captures a comprehensive evaluation of diet quality, providing an understanding of how well chatbot-generated diets align with established nutritional standards. This approach underlines not only the strengths of these tools in areas like dietary variety and adequacy but also uncovers critical gaps, such as their inability to optimise macronutrient balance effectively.

The findings of this study, which demonstrate relatively high total DQI-I scores across all chatbot-generated diet plans (DQI-I > 70), are consistent with the growing body of evidence suggesting that AI-driven tools can achieve satisfactory levels of dietary adequacy and variety. Recent studies have evaluated the potential of AI tools in dietetics, showing promising results in generating nutritionally adequate meal plans [25,26,27,38]. AI-generated diet plans for cardiac patients demonstrated over 75% compliance with dietary guidelines, though some instances of non-compliance were noted [39]. A knowledge-based recommendation framework achieved 92% accuracy in nutrient recommendations across various user groups [40]. For weight management, AI-generated diet plans were often indistinguishable from those created by tertiary medical centres and showed potential for clinical applications, despite some limitations in specificity and affordability [25,41,42]. Similarly, The PROTEIN AI Advisor, a knowledge-based recommendation framework, has been shown to provide highly accurate diet plans spanning across ten user groups, with a total recommendation accuracy of 92% for all nutrient recommendation [40]. Furthermore, AI tools have also been shown to be capable of adapting to individual user profiles, including those with complex dietary needs [15,40]. These findings suggest that AI tools have the potential to revolutionise the field of dietetics, providing personalised and effective meal planning solutions that are tailored to individual users’ needs and preferences [37,43,44].

Macronutrient balance, which includes the distribution of carbohydrates, proteins, and fats, as well as the quality of dietary fats, is essential for an effective diet plan [45,46]. However, the *balance* subscale, which assesses macronutrient and fatty acid ratios, consistently received the lowest scores across all chatbots, highlighting a key area for improvement in AI-driven diet planning tools. A possible explanation for this limitation lies in the fundamental difficulty of programming algorithms to address the complex interactions between macronutrients and the unique dietary needs of individuals. In our study, the chatbots were tasked with generating low-calorie diet plans (1400–1800 kcal), which may have exacerbated their difficulty in achieving optimal macronutrient distribution. Lower-calorie diets inherently pose a challenge, as they require careful allocation of limited energy across all macronutrients while maintaining overall nutritional adequacy. This could partly explain the consistently low scores observed in the balance subscale. In contrast, a study examining meal plans created by ChatGPT 4.0 and Bard for a 25-year-old woman with a higher energy requirement of 2200 kcal reported that both tools generally met the daily DRIs for macronutrients [47]. The analysis revealed that these AI models provided diverse and nutritionally balanced meal options across various dietary patterns, including omnivorous, vegetarian, and vegan diets [47]. This comparison raises questions about the adaptability of AI-driven diet planning tools to different caloric needs and dietary objectives. The findings suggest that while these models may perform well under less restrictive conditions, their effectiveness diminishes when tasked with creating more constrained dietary plans, pointing to a significant limitation in their algorithmic design.

Optimal fatty acid distribution—covering the balance of polyunsaturated (PUFA), monounsaturated (MUFA), and saturated fatty acids (SFA)—is essential for not only energy provision but also vital physiological functions, such as maintaining cell membrane integrity, regulating inflammatory pathways, and supporting cardiovascular health [48,49,50]. The findings of this study reveal significant concerns regarding the macronutrient and fatty acid distribution in chatbot-generated diets. Diets high in saturated fats, trans-fatty acids, and refined carbohydrates, or low in protein and polyunsaturated fatty acids, have been linked to adverse health outcomes including obesity, cardiovascular disease, type 2 diabetes, and certain cancers [51,52]. Furthermore, optimal dietary ratios, including a low linoleic acid to α-linolenic acid (LA/ALA) ratio, have been associated with improved lipid profiles and systemic health outcomes [53]. These findings emphasise the critical role of well-balanced macronutrient and fatty acid profiles in dietary planning [54,55]. The inability of chatbots to effectively address these complex aspects of fatty acid and macronutrient balances represent a significant limitation in their current design. Relying on these diets without proper evaluation may increase the risk of developing diet-related health issues. Therefore, professional nutritional guidance is crucial to reduce these risks.

The finding that diet plans for females scored significantly higher in both “*variety—food groups*” and “*variety—protein sources*” sub-scores compared to those for males raises important questions about the design and training of chatbot algorithms. This disparity could arise from algorithmic biases informed by societal norms or training datasets that emphasise greater dietary diversity for women [56,57], possibly due to their unique nutritional requirements during reproductive years or broader dietary trends [58]. Such biases, while unintentional, underscore the need for more inclusive and balanced datasets to ensure equitable dietary recommendations for both genders. These results are consistent with previous studies suggesting that women tend to prioritise food variety more than men, potentially due to health awareness campaigns targeting specific micronutrient deficiencies [59,60]. Future research should explore whether these gender-based differences continue across various dietary patterns and caloric ranges, ensuring AI algorithms can provide equally comprehensive and unbiased nutrition plans for both males and females.

In terms of energy content of chatbots-generated diet plans, ChatGPT 4.0 demonstrated higher precision in meeting requested caloric targets compared to other chatbots, with none of its diet plans deviating by more than or equal to 20% from the specified calorie level. In contrast, 50% of the diet plans generated by Gemini exceeded the target by over 20%, highlighting a significant limitation in its algorithm’s ability to adhere to caloric constraints. This can be due to several factors, including the lack of personalisation and the inability to fully understand the user’s needs and preferences, as well as algorithmic errors in accurately determining the calorie content of foods [61,62]. Recent studies found that ChatGPT’s recommended daily caloric intakes deviated from the target energy intake, with differences up to 20% [63,64,65]. However, when the target’s energy intake was specifically requested in the prompt, there was a significant reduction in caloric deviations from the optimal energy intake. Another study found that ChatGPT’s ability to provide tailored dietary advice was adequate, but it was unable to consistently exhibit accuracy in delivering tailored dietary advice or plans, especially in complex situations necessitating customised strategies [66]. Nevertheless, chatbots can still be improved to provide more personalised and accurate diet plans [67]. For example, a study proposed a three-turn iterative prompting approach to enhance the quality of food effect summarisation and provide more targeted meal plans [68]. Another study suggested that incorporating user feedback and adjusting the chatbot’s responses based on individual needs could improve the accuracy of diet plans [69]. Future research should explore the factors contributing to these inconsistencies and focus on optimising algorithms in underperforming chatbots to improve their practical utility.

Chatbot-generated diet plans provided distinct patterns and limitations in their recommendations, extending beyond the DQI assessment. Regarding meal structure, ChatGPT 4.0 and Microsoft Copilot designed plans with three main meals and three snacks, while Gemini included three main meals and only two snacks. All diet plans included yoghurt, with ChatGPT 4.0 specifying non-fat Greek yoghurt and Microsoft Copilot and Gemini suggesting for standard Greek yoghurt. While salads, such as mixed green salads, were consistently featured in all plans, only Gemini offered dressing options, including lemon and balsamic vinaigrette, and mustard, whereas Microsoft Copilot and ChatGPT 4.0 disregarded dressings entirely. This variation in salad dressings highlights differing approaches to enhancing palatability, with Gemini showing more creativity by offering dressing options, unlike Copilot and ChatGPT 4.0. Red meat was absent across all 30 diet plans, and fish options were restricted to salmon or cod, showing a lack of diversity in animal-based protein sources. Also, this may reflect biases in the training data or an overly cautious approach to red meat due to its association with health risks. Similarly, variations in cheese and egg inclusion were notable; Microsoft Copilot included cottage cheese in all diets, Gemini excluded cheese entirely, and ChatGPT 4.0 incorporated cheese in only two plans. For eggs, Gemini excluded them from male-targeted diets, whereas ChatGPT 4.0 consistently included eggs in male diets, and Microsoft Copilot included eggs in all diets regardless of gender. Beverage recommendations were limited across the chatbot-generated diet plans. Microsoft Copilot included coffee in only one diet plan, while ChatGPT 4.0 did not provide any guidance on water intake or other beverages. In contrast, Gemini offered a general hydration reminder as a supplementary note alongside the diet plan, addressing the importance of maintaining adequate fluid intake. Given the critical role of hydration in overall health and its consistent inclusion in diet plans created by dietitians, this omission stresses a significant gap that needs to be addressed in AI-generated diet plans. As chatbot technology advances, integrating a broader understanding of diverse dietary needs and preferences will be essential for creating more effective and appealing meal plans.

This study offers several notable strengths, providing valuable insights into the capabilities and limitations of AI-driven diet planning tools. One key strength is the quantitative assessment of diet quality using the DQI-I, a comprehensive tool that evaluates variety, adequacy, moderation, and balance across diets. This approach provides a framework for systematically comparing the nutritional quality of chatbot-generated diet plans, an area previously explored primarily through qualitative evaluations involving dietitians. Additionally, this study evaluates multiple chatbots, including ChatGPT 4.0, Microsoft Copilot, and Gemini, across gender-specific and low-calorie dietary scenarios, enabling an understanding of their performance under varying conditions. Despite these strengths, the study has certain limitations. First, while the DQI-I provides a comprehensive measure of diet quality, it does not fully capture other important aspects, including cultural appropriateness or individual dietary preferences, which could influence the acceptability and long-term adherence to AI-generated meal plans. Second, the study focused exclusively on weight-loss diets within a specific calorie range (1400–1800 kcal), limiting the generalisability of findings to higher-calorie or maintenance diets. Lastly, our study focused on evaluating the first response generated by each chatbot, acknowledging the known issue of variability in AI-generated outputs. Despite efforts to standardise the setup and use new user accounts for interactions, it is well recognised that identical prompts can produce varying responses. This inherent variability raises concerns about the reproducibility and reliability of our findings. Exploring strategies to better account for and address this variability, such as analysing multiple responses or assessing consistency across repeated interactions, may be a focus for future studies.

Future research should build upon the current findings to address the identified limitations and explore new dimensions of AI-driven diet planning tools. One promising approach is to periodically replicate this study by generating new diet plans as AI technologies have rapid self-learning and updating capabilities. Additionally, future studies could examine the cultural appropriateness of chatbot-generated diet plans. This would involve evaluating how well these tools adapt to dietary habits, food availability, and cultural preferences across diverse populations. Future research should also incorporate anthropometric parameters such as weight, height, and BMI to evaluate not only the overall diet quality using indices like the DQI-I but also the appropriateness of AI-generated diet plans in meeting individualised energy and nutrient requirements, including protein intake and energy balance. Moreover, expanding the scope of evaluation to include higher-calorie diets, maintenance diets, or diets for specific health conditions could provide a broader understanding of the applicability of these tools in varied contexts. Assessing not only the nutritional metrics but also the feasibility, palatability, and user satisfaction of AI-generated meal plans would offer a more comprehensive evaluation of their utility.

Despite the advancements in AI, the expertise and refined judgement of dietetic professionals remain indispensable, particularly when addressing the complexity of individualised dietary needs. While AI tools excel at automating tasks like calorie calculations and meal diversity, they often fall short in areas requiring deep contextual understanding, such as cultural food preferences, religious dietary restrictions, and individual medical histories. These factors are critical for ensuring patient adherence and satisfaction. Additionally, clinical conditions including metabolic diseases, diabetes, food allergies, and gastrointestinal disorders demand a level of precision and adaptability that current AI models are not yet equipped to provide. While AI can complement the work of dietitians by streamlining routine tasks and providing preliminary assessments, the irreplaceable value of dietetic professionals lies in their ability to synthesise complex information, empathise with patients, and design interventions that are both evidence-based and personalised. Consequently, the integration of AI tools should be viewed as a means to enhance, rather than replace, the critical human element in effective dietary care.

## 5. Conclusions

This study demonstrates the promising potential of AI-driven chatbots in generating nutritionally adequate and varied diet plans, as evidenced by high total DQI-I scores across all evaluated tools. By employing a quantitative framework like the DQI-I, this research provides a standardised reference for assessing the nutritional quality of chatbot-generated diets, marking a critical step forward from previous subjective assessments. Despite their strengths in dietary variety, the chatbots revealed notable gaps, particularly in achieving macronutrient balance and fatty acid distribution. These limitations highlight the challenges of programming algorithms to account for the complex interplay of dietary components, especially in low-calorie scenarios. Additionally, observed gender-based differences and variations in meal structure suggest underlying biases and inconsistencies that need further investigation. As AI technologies evolve, future efforts should focus on enhancing algorithmic complexity to optimise nutritional quality, cultural adaptability, and user personalisation. While advancements in AI-driven tools could position chatbots as transformative assets in personalised nutrition and weight management—supporting diverse dietary needs with greater precision and inclusivity—it is essential to acknowledge the expertise and refined judgement of dietetic professionals. AI can complement, but not replace, the critical role of human professionals in delivering tailored, culturally appropriate, and clinically informed dietary interventions. Future efforts should aim to enhance the collaborative relationship between AI capabilities and professional expertise, ensuring comprehensive and effective dietetics solutions.

## Figures and Tables

**Table 1:** Definition and scoring overview of DQI-I components [21].

Diet Quality Component	Grouping of Diet Quality Component	Scoring Criteria	Score
Variety—food groups	5 food groups: meat/poultry/fish/egg, dairy/beans, grains, fruits, and vegetables	Each food group awarded 0 or 3 pts: 3 points awarded if at least 1 item from that group was consumed	0–15
Variety—protein sources	6 sources: meat, poultry, fish, dairy, beans, eggs	3 or more sources consumed: 5 pts 2 sources consumed: 3 pts 1 source consumed: 1 pts 0 sources consumed: 0 pts	0–5
Adequacy	8 groups: vegetables, fruit, grain, fibre, protein, iron, calcium, vitamin C	Between 0 and 5 points awarded for each of the 8 adequacy groups, depending on percentage of Recommended Daily Allowance (RDA) met	0–40
Moderation	6 groups: total fat, saturated fat, cholesterol, sodium, empty calorie foods	Between 0 and 6 points awarded for each of the 5 moderation groups, depending on percentage of RDA met	0–30
Balance	2 groups: macronutrient ratio, fatty acid ratio, fatty acid ratio	Between 0 and 6 points awarded depending on ratio of macronutrients, and between 0 and 4 points awarded depending on ratio of fatty acids	0–10

**Table 2:** Mean and standard deviation of total DQI-I scores and sub-scores for Gemini, Microsoft Copilot, ChatGPT 4.0, and overall.

Chatbot	Variety—Food Groups	Variety—Protein Sources	Adequacy	Moderation	Balance	Total DQI-I Score
Gemini (*n* = 10)	15.00 (±0.0)	4.60 (±0.8)	33.00 (±1.8)	18.90 (±4.2)	0.40 (±1.3)	71.90 (±4.1)
Microsoft Copilot (*n* = 10)	15.00 (±0.0)	5.00 (±0.0)	34.50 (±2.1)	17.40 (±2.4)	0.40 (±0.8)	72.30 (±4.1)
ChatGPT 4.0 (*n* = 10)	14.70 (±0.9)	5.00 (±0.0)	34.70 (±2.1)	16.80 (±3.5)	00.00 (±0.0)	71.20 (±5.2)
Overall (*n* = 30)	14.90 (±0.5)	4.87 (±0.5)	34.07 (±2.1)	17.70 (±3.5)	0.27 (±0.9)	71.80 (±4.3)

**Table 3:** Mean and standard deviation of total DQI-I scores and sub-scores for diet plans generated for females (*n* = 15) and males (*n* = 15).

Gender	Variety—Food Groups	Variety—Protein Sources	Adequacy	Moderation	Balance	Total DQI-I Score
Female (*n* = 15)	15.00 (±0.0)	5.00 (±0.0)	34.27 (±1.9)	17.20 (±3.8)	0.27 (±1.3)	71.73 (±3.9)
Male (*n* = 15)	14.80 (±0.7)	4.73 (±0.7)	33.87 (±2.3)	18.20 (±3.1)	0.27 (±0.7)	71.87 (±4.9)
*p* value *	0.040 **	0.002 **	0.579	0.654	0.895	0.561

* The *p*-value from the independent-sample t-test was used to determine whether there were statistically significant differences in total DQI-I scores and sub-scores between females and males. ** Statistically significant differences between females and males.

**Table 4:** Number and percentage of diet plans categorised by percentage differences between requested calorie levels and generated calorie content by chatbots.

Chatbot	<5%	5–9.99%	10–14.99%	15–19.99%	≥20%
Gemini (*n* = 10)	*n* = 2 (20%)	*n* = 0 (0%)	*n* = 1 (10%)	*n* = 2 (20%)	*n* = 5 (50%)
Microsoft Copilot (*n* = 10)	*n* = 3 (30%)	*n* = 2 (20%)	*n* = 2 (20%)	*n* = 2 (20%)	*n* = 1 (10%)
ChatGPT 4.0 (*n* = 10)	*n* = 2 (20%)	*n* = 5 (50%)	*n* = 2 (20%)	*n* = 1 (10%)	*n* = 0 (0%)

## Data Availability

Full data supporting the results and conclusions of this article will be made available by the authors on request.

## Supplementary Materials

The following supporting information can be downloaded at: https://www.mdpi.com/article/10.3390/nu17020206/s1.
