# Supplementary material for: Diet Quality and Caloric Accuracy in AI-Generated Diet Plans: A Comparative Study Across Chatbots
Source: Nutrients. 2025 Jan 7;17(2):206. doi: 10.3390/nu17020206 (PMC11768065; doi:10.3390/nu17020206)
Supplement: Supplementary file 1 [file nutrients-17-00206-s001.zip › nutrients-3391735-supplementary.pdf]

This supplementary document provides additional details and examples to support the findings of our study on the diet plans generated by chatbots and their assessment using the Diet Quality Index-International (DQI-I). The content included in this document aims to enhance the transparency and reproducibility of the research process.

Specifically, the document contains:

1. **Some examples of Chatbot-Generated Weight Loss Diet Plans**: Sample diet plans of varying calorie levels, illustrating the output provided by different chatbot models.
2. **An example of calculation of DQI-I Score**: Step-by-step breakdown of the scoring methodology applied to evaluate the overall diet quality of each plan, along with the derived sub-scores.

ChatGPT 4.0

Here's a **1,500**-calorie daily meal plan for a 35-year-old **female**, designed to support weight loss with balanced macronutrients and specified portion sizes in grams.

Meal Plan Summary:

- **Calories:** 1,500 kcal
- **Macronutrient Breakdown:**
  - Protein: ~25%
  - Carbohydrates: ~40%
  - Fats: ~35%

Breakfast

- **Avocado Toast with Eggs**
  - Whole-grain bread: 1 slice (40g) (80 kcal)
  - Avocado: 50g (80 kcal)
  - Egg (poached or boiled): 1 large (50g) (70 kcal)
  - Spinach (sautéed): 30g (5 kcal)
  - **Total:** 235 kcal

Mid-Morning Snack

- **Greek Yogurt and Berries**
  - Greek yogurt (non-fat): 150g (90 kcal)
  - Blueberries: 50g (30 kcal)
  - Chia seeds: 5g (25 kcal)
  - **Total:** 145 kcal

Lunch

- **Grilled Chicken and Quinoa Salad**
  - Grilled chicken breast: 100g (165 kcal)
  - Quinoa (cooked): 80g (110 kcal)
  - Mixed greens (lettuce, spinach): 80g (15 kcal)
  - Cherry tomatoes: 50g (10 kcal)
  - Cucumber: 50g (5 kcal)
  - Red bell pepper: 30g (10 kcal)
  - Olive oil: 10g (90 kcal)
  - **Total:** 405 kcal

Afternoon Snack

- **Apple with Almonds**
  - Apple: 100g (50 kcal)
  - Almonds: 15g (90 kcal)
  - **Total:** 140 kcal

Dinner

- **Baked Salmon with Steamed Broccoli and Sweet Potato**
  - Salmon fillet: 100g (200 kcal)
  - Sweet potato (baked): 100g (85 kcal)
  - Broccoli (steamed): 80g (25 kcal)
  - Olive oil: 5g (45 kcal)
  - **Total:** 355 kcal

Evening Snack

- **Cottage Cheese and Dark Chocolate**
  - Cottage cheese (low-fat): 100g (90 kcal)
  - Dark chocolate (70-85%): 10g (50 kcal)
  - **Total:** 140 kcal

Daily Totals:

- **Calories:** 1,500 kcal
- **Protein:** ~25% (approx. 94g)
- **Carbohydrates:** ~40% (approx. 150g)
- **Fats:** ~35% (approx. 58g)

Here's a **1,700**-calorie daily meal plan for a 35-year-old **female**, designed to support weight loss with balanced macronutrients and portion sizes in grams.

Meal Plan Summary:

- **Calories:** 1,700 kcal
- **Macronutrient Breakdown:**
  - Protein: ~25%
  - Carbohydrates: ~40-45%
  - Fats: ~30-35%

Breakfast

- **Avocado Toast with Cottage Cheese and Berries**
  - Whole-grain bread: 1 slice (40g) (80 kcal)
  - Avocado: 50g (80 kcal)
  - Cottage cheese (low-fat): 100g (90 kcal)
  - Blueberries: 50g (30 kcal)
  - **Total:** 280 kcal

Mid-Morning Snack

- **Greek Yogurt and Almonds**
  - Greek yogurt (non-fat): 150g (90 kcal)
  - Almonds: 15g (90 kcal)
  - **Total:** 180 kcal

Lunch

- **Turkey and Quinoa Salad**
  - Turkey breast (grilled, skinless): 120g (165 kcal)
  - Quinoa (cooked): 100g (125 kcal)
  - Mixed greens (lettuce, spinach): 80g (15 kcal)
  - Cherry tomatoes: 50g (10 kcal)
  - Cucumber: 50g (5 kcal)
  - Red bell pepper: 30g (10 kcal)
  - Olive oil: 10g (90 kcal)
  - **Total:** 420 kcal

Afternoon Snack

- **Apple with Peanut Butter**
  - Apple: 120g (60 kcal)
  - Peanut butter (natural): 15g (90 kcal)
  - **Total:** 150 kcal

Dinner

- **Grilled Salmon with Sweet Potato and Steamed Asparagus**
  - Salmon fillet: 120g (240 kcal)
  - Sweet potato (baked): 120g (100 kcal)
  - Asparagus (steamed): 80g (20 kcal)
  - Olive oil: 5g (45 kcal)
  - **Total:** 405 kcal

Evening Snack

- **Dark Chocolate and Mixed Berries**
  - Dark chocolate (70-85%): 10g (50 kcal)
  - Strawberries: 50g (15 kcal)
  - Raspberries: 50g (25 kcal)
  - **Total:** 90 kcal

Daily Totals:

- **Calories:** 1,700 kcal
- **Protein:** ~25% (approx. 107g)
- **Carbohydrates:** ~40-45% (approx. 170g)
- **Fats:** ~30% (approx. 63g)

Here's a **1,400**-calorie daily meal plan designed for weight loss for a 35-year-old **male**. It includes a balance of protein, fats, and carbohydrates with portion sizes in grams.

Meal Plan Summary:

- **Calories:** 1,400 kcal
- **Macronutrient Breakdown:**
    - Protein: ~25-30%
    - Carbohydrates: ~35-40%
    - Fats: ~30-35%

Breakfast

- **Greek Yogurt with Berries and Nuts**
    - Greek yogurt (non-fat): 150g (90 kcal)
    - Strawberries: 50g (15 kcal)
    - Almonds: 10g (60 kcal)
    - Honey: 5g (15 kcal)
    - **Total:** 180 kcal

Mid-Morning Snack

- **Hard-Boiled Eggs**
    - Eggs: 2 large (100g) (140 kcal)
    - **Total:** 140 kcal

Lunch

- **Turkey Salad with Avocado and Olive Oil**
    - Turkey breast (grilled, skinless): 100g (135 kcal)
    - Mixed greens (lettuce, spinach): 80g (15 kcal)
    - Cherry tomatoes: 50g (10 kcal)
    - Cucumber: 50g (5 kcal)
    - Red bell pepper: 30g (10 kcal)
    - Avocado: 30g (50 kcal)
    - Olive oil: 10g (90 kcal)
    - **Total:** 315 kcal

Afternoon Snack

- **Apple and Almond Butter**
    - Apple: 100g (50 kcal)
    - Almond butter (natural): 10g (60 kcal)
    - **Total:** 110 kcal

Dinner

- **Grilled Cod with Sweet Potato and Steamed Vegetables**
    - Cod fillet: 120g (110 kcal)
    - Sweet potato (baked): 100g (85 kcal)
    - Zucchini (steamed): 50g (10 kcal)
    - Green beans (steamed): 50g (15 kcal)
    - Olive oil: 5g (45 kcal)
    - **Total:** 265 kcal

Evening Snack

- **Cottage Cheese and Blueberries**
    - Cottage cheese (low-fat): 100g (90 kcal)
    - Blueberries: 50g (30 kcal)
    - **Total:** 120 kcal

Daily Totals:

- **Calories:** 1,400 kcal
- **Protein:** ~30% (approx. 105g)
- **Carbohydrates:** ~35-40% (approx. 140g)
- **Fats:** ~30% (approx. 47g)

Here’s an **1,800**-calorie daily meal plan for a 35-year-old **male**, structured to support weight loss with balanced macronutrients and portion sizes in grams.

Meal Plan Summary:

- **Calories:** 1,800 kcal
- **Macronutrient Breakdown:**
  - Protein: ~25%
  - Carbohydrates: ~40-45%
  - Fats: ~30-35%

Breakfast

- **Oatmeal with Almonds and Berries**
  - Rolled oats: 50g (190 kcal)
  - Almond milk (unsweetened): 150 ml (25 kcal)
  - Blueberries: 50g (30 kcal)
  - Almonds: 10g (60 kcal)
  - **Total:** 305 kcal

Mid-Morning Snack

- **Greek Yogurt with Honey and Walnuts**
  - Greek yogurt (non-fat): 150g (90 kcal)
  - Honey: 5g (15 kcal)
  - Walnuts: 10g (65 kcal)
  - **Total:** 170 kcal

Lunch

- **Turkey and Brown Rice Salad**
  - Turkey breast (grilled, skinless): 120g (165 kcal)
  - Brown rice (cooked): 100g (110 kcal)
  - Mixed greens (lettuce, spinach): 80g (15 kcal)
  - Cherry tomatoes: 50g (10 kcal)
  - Red bell pepper: 30g (10 kcal)
  - Cucumber: 50g (5 kcal)
  - Olive oil: 10g (90 kcal)
  - **Total:** 405 kcal

Afternoon Snack

- **Apple with Peanut Butter**
  - Apple: 120g (60 kcal)
  - Peanut butter (natural): 15g (90 kcal)
  - **Total:** 150 kcal

Dinner

- **Grilled Salmon with Quinoa and Steamed Vegetables**
  - Salmon fillet: 120g (240 kcal)
  - Quinoa (cooked): 80g (110 kcal)
  - Broccoli (steamed): 80g (25 kcal)
  - Zucchini (steamed): 50g (10 kcal)
  - Olive oil: 5g (45 kcal)
  - **Total:** 430 kcal

Evening Snack

- **Cottage Cheese with Strawberries and Dark Chocolate**
  - Cottage cheese (low-fat): 100g (90 kcal)
  - Strawberries: 50g (15 kcal)
  - Dark chocolate (70-85%): 10g (50 kcal)
  - **Total:** 155 kcal

Daily Totals:

- **Calories:** 1,800 kcal
- **Protein:** ~25% (approx. 110g)
- **Carbohydrates:** ~40-45% (approx. 170g)
- **Fats:** ~30% (approx. 67g)

Microsoft Copilot

Alright! Here's a **1600** kcal daily meal plan for a 35-year-old **woman**:

Breakfast:

- Scrambled eggs (2 large eggs, 100g)
- Spinach (50g)
- Cherry tomatoes (100g)
- 1 slice of whole-grain toast (30g)

Morning Snack:

- Greek yogurt (150g) with a few strawberries (50g)

Lunch:

- Grilled chicken breast (120g)
- Mixed salad (lettuce, cucumber, bell pepper, carrots) with lemon vinaigrette (200g total)
- Brown rice (90g cooked)

Afternoon Snack:

- Cottage cheese (100g) with sliced bell peppers (100g)

Dinner:

- Baked cod (130g)
- Quinoa (70g cooked)
- Steamed green beans (100g)

Evening Snack:

- A small apple (100g) and 10 almonds (15g)

Sure thing! Here's a **1700** kcal daily meal plan for a 35-year-old **woman**:

Breakfast:

- Greek yogurt (200g) with mixed berries (100g) and a tablespoon of chia seeds (10g)
- 1 slice of whole-grain toast (30g) with avocado (50g)

Morning Snack:

- 1 hard-boiled egg (50g)
- A small apple (100g)

Lunch:

- Grilled chicken breast (130g)
- Mixed salad (lettuce, cucumber, cherry tomatoes) with olive oil and lemon dressing (200g total)
- Brown rice (100g cooked)

Afternoon Snack:

- Cottage cheese (100g) with carrot sticks (100g)

Dinner:

- Baked salmon (130g)
- Quinoa (80g cooked)
- Steamed green beans (100g)

Evening Snack:

- Greek yogurt (100g) with a few blueberries (50g)
- 10 almonds (15g)

Got it! Here’s a **1600** kcal daily meal plan for a 35-year-old **man**:

Breakfast:

- Scrambled eggs (2 large eggs, 100g)
- Spinach (50g)
- Cherry tomatoes (100g)
- 1 slice of whole-grain toast (30g)

Morning Snack:

- Greek yogurt (150g) with a few blueberries (50g)

Lunch:

- Grilled chicken breast (140g)
- Mixed salad (lettuce, cucumber, bell pepper, carrots) with olive oil and lemon dressing (200g total)
- Brown rice (80g cooked)

Afternoon Snack:

- Cottage cheese (100g) with carrot sticks (100g)

Dinner:

- Baked cod (130g)
- Quinoa (70g cooked)
- Steamed broccoli (100g)

Evening Snack:

- A small apple (100g) and 10 almonds (15g)

Here's a **1700** kcal meal plan for a 35-year-old **man** aiming to lose weight:

Breakfast:

- Oatmeal (50g) cooked with water
- Half a banana (60g)
- 1 tablespoon of peanut butter (16g)

Morning Snack:

- Greek yogurt (150g) with a few strawberries (50g)

Lunch:

- Grilled chicken breast (150g)
- Mixed salad (lettuce, cucumber, cherry tomatoes) with olive oil and lemon dressing (200g total)
- Brown rice (90g cooked)

Afternoon Snack:

- Cottage cheese (100g) with carrot sticks (100g)

Dinner:

- Baked salmon (150g)
- Quinoa (80g cooked)
- Steamed broccoli (100g)

Evening Snack:

- A small apple (100g) and 10 almonds (15g)

Gemini

This is for informational purposes only. For medical advice or diagnosis, consult a professional.

Absolutely! Here's a sample **1400**-calorie meal plan for a 35-year-old **woman**, incorporating specific food names and portion sizes:

1400 Calorie Meal Plan for a 35-Year-Old Female

Breakfast (300 calories)

- Oatmeal (40g) with 100g blueberries and 15g chopped walnut

Snack 1 (200 calories)

- A medium size banana with 20g almonds

Lunch (300 calories)

- Salad with mixed greens (100g), 100g grilled chicken, 50g chickpeas, 50g cherry tomatoes, and 20g balsamic vinaigrette

Snack 2 (200 calories)

- Greek yogurt (150g) with 20g granola

Dinner (400 calories)

- Grilled salmon (120g) with 150g roasted sweet potatoes and 150g steamed asparagus

Tips:

- **Hydrate:** Drink plenty of water throughout the day.
- **Prioritize Protein:** Include protein in every meal to keep you feeling full and satisfied.
- **Choose Whole Grains:** Opt for whole-grain bread, pasta, and rice for sustained energy.
- **Limit Processed Foods:** Focus on whole, unprocessed foods.
- **Listen to Your Body:** Pay attention to hunger and fullness cues.

Recipe for Oatmeal with Blueberries and Walnuts

Ingredients:

- 40g rolled oats
- 250ml water
- 100g blueberries
- 15g chopped walnuts

Instructions:

1. Bring water to a boil in a saucepan.
2. Add oats and reduce heat to low.
3. Simmer for 5 minutes, stirring occasionally.
4. Remove from heat and stir in blueberries and walnuts.

Recipe for Grilled Salmon with Roasted Sweet Potatoes and Asparagus

Ingredients:

- 120g salmon fillet
- 150g sweet potatoes, peeled and diced
- 150g asparagus
- 1 tablespoon olive oil

Instructions:

1. Preheat grill to medium-high heat.
2. Season salmon with salt and pepper.
3. Grill salmon for 4-5 minutes per side, or until cooked through. 4. Toss sweet potatoes and asparagus with olive oil, salt, and pepper.
4. Roast vegetables in a preheated oven at 200°C for 20-25 minutes, or until tender.

Remember: This is a general meal plan, and you may need to adjust it based on your individual needs and preferences. If you have any underlying health conditions or dietary restrictions, please consult with a registered dietitian or healthcare professional to create a personalized meal plan.

This is for informational purposes only. For medical advice or diagnosis, consult a professional.

Absolutely! Here's a sample **1500**-calorie meal plan for a 35-year-old **woman**, incorporating specific food names and portion sizes:

1500 Calorie Meal Plan for a 35-Year-Old Female

Breakfast (350 calories)

- Greek yogurt (200g) with 120g raspberries and a drizzle of honey (10g)

Snack 1 (200 calories)

- A medium size apple with 20g walnuts

Lunch (400 calories)

- Whole-wheat sandwich with 70g turkey or lean ham, lettuce (20g), tomato (20g), and mustard

Snack 2 (200 calories)

- Greek yogurt (150g) with 20g granola

Dinner (350 calories)

- Lentil soup (300ml) with lettuce salad (150g) and a whole-wheat roll (50g)

Tips:

- **Hydrate:** Drink plenty of water throughout the day.
- **Prioritize Protein:** Include protein in every meal to keep you feeling full and satisfied.
- **Choose Whole Grains:** Opt for whole-grain bread, pasta, and rice for sustained energy.
- **Limit Processed Foods:** Focus on whole, unprocessed foods.
- **Listen to Your Body:** Pay attention to hunger and fullness cues.

Recipe for Lentil Soup

Instructions:

- Rinse lentils under cold water.
- In a large pot, combine lentils, vegetable broth, onion, carrots, celery, garlic, and bay leaf.
- Bring to a boil, then reduce heat and simmer for 30-40 minutes, or until lentils are tender.
- Season with salt and pepper to taste.

Recipe for whole-Wheat Sandwich

Ingredients:

- 2 slices whole-wheat bread
- 70g turkey or lean ham
- 20g lettuce
- 20g tomato
- Mustard (10g)

Instructions:

1. Prepare the bread: Toast the bread slices to your desired level of crispiness.
2. Assemble the sandwich:
3. Spread mustard on one slice of bread.
4. Layer the turkey or ham on top of the mustard.
5. Add the lettuce and tomato slices.
6. Top with the other slice of bread.

Tip: For extra flavor, you can add other condiments like mayonnaise, avocado, or a drizzle of olive oil.

Remember: This is a general meal plan, and you may need to adjust it based on your individual needs and preferences. If you have any underlying health conditions or dietary restrictions, please consult with a registered dietitian or healthcare professional to create a personalized meal plan

This is for informational purposes only. For medical advice or diagnosis, consult a professional.

Absolutely! Here's a sample **1500**-calorie meal plan for a 35-year-old **man**, incorporating specific food names and portion sizes:

1500 Calorie Meal Plan for a 35-Year-Old Male

Breakfast (350 calories)

- Oatmeal (50g) with 150g raspberries and 15g chopped walnut

Snack 1 (200 calories)

- Trail mix (75g) nuts, seeds and dried fruit

Lunch (400 calories)

- Whole-wheat sandwich with 75g turkey or lean ham, lettuce (50g), tomato (50g), and mustard

Snack 2 (200 calories)

- Greek yogurt (200g) with honey(1tablespoon/21g)

Dinner (350 calories)

- Chicken Stir-fry (150g) and a variety of colorful vegetables 150g broccoli, bell peppers, snow peas) over 100g cooked brown rice

Tips:

- **Hydrate:** Drink plenty of water throughout the day.
- **Prioritize Protein:** Include protein in every meal to keep you feeling full and satisfied.
- **Choose Whole Grains:** Opt for whole-grain bread, pasta, and rice for sustained energy.
- **Limit Processed Foods:** Focus on whole, unprocessed foods.
- **Listen to Your Body:** Pay attention to hunger and fullness cues.

Recipe for whole-Wheat Sandwich

Ingredients:

- 2 slices whole-wheat bread
- 75g turkey or lean ham
- 50g lettuce
- 50g tomato
- Mustard (10g)

Recipe for chicken and a variety of colorful vegetables over cooked brown rice

Ingredients:

- 150g boneless, skinless chicken breast
- 150g mixed vegetables (broccoli 50g, bell peppers 50g , snow peas 50g)
- 100 g brown rice
- 1 tablespoons soy sauce
- 1 teaspoon sesame oil

Instructions:

1. Cook the rice: Cook the brown rice according to package instructions.
2. Prepare the vegetables: Cut the vegetables into bite-sized pieces.
3. Cook the protein:

   Chicken breast: Cut the breast into cubes and press to remove excess water. Stir-fry until golden brown.
4. Stir-fry the vegetables: Heat a wok or large skillet over high heat with a little oil. Stir-fry for 30 seconds, then add the vegetables. Stir-fry until crisp-tender.
5. Serve: Serve the stir-fry over the cooked brown rice.

Remember: This is a general meal plan, and you may need to adjust it based on your individual needs and preferences. If you have any underlying health conditions or dietary restrictions, please consult with a registered dietitian or healthcare professional to create a personalized meal plan

This is for informational purposes only. For medical advice or diagnosis, consult a professional.

Absolutely! Here's a sample **1700**-calorie meal plan for a 35-year-old **man**, incorporating specific food names and portion sizes:

1700 Calorie Meal Plan for a 35-Year-Old Male

Breakfast (400 calories)

- Oatmeal (60g) with 150g blueberries and 20g chopped walnut

Snack 1 (200 calories)

- Greek yogurt (250g) with honey(1tablespoon/21g)

Lunch (450 calories)

- Salad with mixed greens (150g), 200g grilled chicken, 60g cherry tomatoes, and 20g balsamic vinaigrette

Snack 2 (200 calories)

- 200g raspberry with 20g almond butter

Dinner (450 calories)

- Chicken Stir-fry (200g) and a variety of colorful vegetables 150g broccoli, bell peppers, snow peas) over 125g cooked brown rice

Tips:

- **Hydrate:** Drink plenty of water throughout the day.
- **Prioritize Protein:** Include protein in every meal to keep you feeling full and satisfied.
- **Choose Whole Grains:** Opt for whole-grain bread, pasta, and rice for sustained energy.
- **Limit Processed Foods:** Focus on whole, unprocessed foods.
- **Listen to Your Body:** Pay attention to hunger and fullness cues.

Recipe for chicken and a variety of colorful vegetables over cooked brown rice

Ingredients:

- 200g boneless, skinless chicken breast
- 150g mixed vegetables (broccoli 50g, bell peppers 50g , snow peas 50g)
- 125 g brown rice
- 2 tablespoons soy sauce
- 1 teaspoon sesame oil

Instructions:

1. Cook the rice: Cook the brown rice according to package instructions.
2. Prepare the vegetables: Cut the vegetables into bite-sized pieces.
3. Cook the protein:
    a. Chicken breast: Cut the breats into cubes and press to remove excess water. Stir-fry until golden brown.
4. Stir-fry the vegetables: Heat a wok or large skillet over high heat with a little oil. Stir-fry for 30 seconds, then add the vegetables. Stir-fry until crisp-tender.
5. Combine: Add the cooked protein to the vegetables and stir-fry for a few more minutes to combine.
6. Serve: Serve the stir-fry over the cooked brown rice.

Remember: This is a general meal plan, and you may need to adjust it based on your individual needs and preferences. If you have any underlying health conditions or dietary restrictions, please consult with a registered dietitian or healthcare professional to create a personalized meal plan.

1400 KCAL DIET PLAN (Female) - ChatGPT																		
	FOOD	AMOUNT		Fibre (g)	Protein (g)	Iron (mg)	Calcium (mg)	Vitamin C (mg	Sodium (mg)	Total Fat (g)	Sat. Fat (g)	MUFA (g)	PUFA (g)	Cholesterol (r	CHO (g)	Fat	Energy (kcal)	Energy AI (kcal)
BREAKFAST	Oatmeal with Berries and Almond Butter																	
	Rolled oats	40 g		4	5	1.7	20	0	0	2.5	0	1	1	0	27		140	150
	Almond milk (unsweetened)	150 ml		0.3	0.6	0.42	276	0	108	1.44	0.12	0.885	0.36	0	1.965		22.5	25
	Blueberries	50 g		1.2	0.37	0.14	3	4.85	0.5	0.165	0.014	0.0235	0.073	0	7.25		28.5	30
	Almond butter	10 g		1.03	2.096	0.349	34.7	0	22.7	5.55	0.655	3.244	1.361	0	1.882		61.4	60
	Chia seeds	5 g		1.72	0.825	0.386	31.55	0.08	0.8	1.535	0.166	0.115	1.185	0	2.105		24.3	25
LUNCH	Grilled chicken breast	100 g		0	30.5	0.45	5	0	52	3.17	0.992	1.21	0.764	104	0		151	165
	Mixed greens (lettuce, spinach)	80 g		1.12	1.288	1.04	36.8	8.8	28.8	0.176	0.025	0.006	0.087	0	2.344		12.8	15
	Cherry tomatoes	50 g		0.8	0.82	0.59	16.5	4.65	30.5	0	0	0	0	0	4.92		13	10
	Cucumber	50 g		0.25	0.325	0.14	8	1.4	1	0.055	0.018	0.002	0.016	0	1.815		7.5	5
	Red bell pepper	30 g		0.36	0.27	10.5	1.8	42.6	0.72	0.039	0	0	0	0	1.995		8.1	10
	Avocado	30 g		2	0.6	0.165	3.6	3	2.1	4.4	0.64	2.94	0.546	0	2.56		48	50
	Olive oil	10 g		0	0	0.056	0.1	0	0.2	10	1.38	7.29	1.052	0	0		88.4	90
DINNER	Salmon with Steamed Vegetables and Quinoa																	
	Salmon fillet	100 g		0	25.82	0.48	9	0.5	417	5.54	1.021	1.698	1.022	58	0.1		160	200
	Quinoa (cooked)	80 g		2.24	3.52	1.192	13.6	0	5.6	1.536	0.184	0.422	0.864	0	17.04		96	110
	Steamed broccoli	80 g		2.57	1.86	0.523	31.2	50.6	32	0.32	0.062	0.031	0.133	0	5.6		27.3	25
	Carrots (steamed)	50 g		1.5	0.38	0.17	15	1.8	29	0.09	0.015	0.003	0.044	0	4.11		17.5	20
	Olive oil	5 g		0	0	0.028	0.05	0	0.01	5	0.69	3.645	0.526	0	0		44.2	45
SNACK 1	Greek yogurt (non-fat)	100 g		0	10.3	0.07	111	0	36	0.37	0.108	0.043	0.011	5	3.64		59	60
	Walnuts	10 g		0.67	1.52	0.291	9.8	0.13	0.2	6.52	0.613	0.893	4.72	0	1.37		65.4	65
SNACK 2	Apple	100 g		2.4	0.26	0.12	6	4.6	1	0.17	0.028	0.007	0.051	0	13.8		52	50
	Peanut Butter (natural)	10 g		0.48	2.25	0.173	4.9	0	42.9	5.11	1.01	2.54	1.23	0	2.23		59.7	60
SNACK 3	Cottage cheese (low-fat)	100 g		0	11	0.13	103	0	321	2.3	1.26	0.469	0.072	12	4.31		82	90
	Dark chocolate (70-85%)	10 g		1.09	0.779	1.19	7.3	0	2	4.26	2.45	1.28	0.126	0.3	4.59		59.8	50
			Total:	23.73	100.383	20.303	747.9	123.01	1134.03	60.246	11.451	27.7465	15.243	179.3	110.626		1328.4	1410
			Macronutrient Cal		401.532					542.214	103.059				442.504		1386.25	
			Macronutrient%		28.9653381					39.1137241	7.75813008				31.9209378			
			RDA	19.6		18	1000	75	2300								empty calori percentage	
			RDA%	121.07	10-35	112.79	74.79	164.013333		20-35	10				45-65		4.27142857	
										P/S	1.33115012	M/S	2.42306349					

Variety - food	Score	Variety - protei	Score	Adequacy	Score	Moderation	Score	Balance	Score	Total
meat/poultry/	3	meat		vegetables	5	total fat	0	macronutrien	0	71
dairy/beans	3	poultry	x	fruit	3	saturated fat	3	fatty acid ratio	0	
grains	3	fish	x	grain	2	cholesterol	6	Total	0	
fruits	3	dairy	x	fiber	5	sodium	6			
vegetables	3	beans		protein	5	empty calorie	3			
Total	15	eggs		iron	5	Total	18			
		total	5	calcium	3					
				vitamin C	5					
				Total	33					

Diet plan	Variety-food groups					Variety-protein sources	Adequacy								Moderation					Balance				
	meat/poultry/fish/egg	dairy/beans	grains	fruits	vegetables	meat / poultry / fish / dairy / beans / eggs	vegetables	fruit	grain	fiber	protein	iron	calcium	vitamin C	total fat	saturated fat	cholesterol	sodium	empty calorie foods	macronutrient ratio	fatty acid ratio	TOTAL SCORE	requested diet calorie	actual calorie
Gemini 1400 F	3	3	3	3	3	5	5	5	3	5	5	3	2	5	0	6	6	6	6	0	0	77	1400	1732
Gemini 1500 F	3	3	3	3	3	5	3	5	3	5	5	3	3	4	0	3	6	3	6	0	4	73	1500	1449
Gemini 1600 F	3	3	3	3	3	5	5	2	3	5	5	5	5	5	0	3	0	3	6	0	0	67	1600	2131
Gemini 1700 F	3	3	3	3	3	5	5	5	3	5	5	3	3	5	0	6	6	6	6	0	0	78	1700	2038
Gemini 1800 F	3	3	3	3	3	5	4	2	5	5	5	3	3	5	0	3	0	3	6	0	0	64	1800	1864
Gemini 1400 M	3	3	3	3	3	5	5	5	2	5	5	5	2	3	0	3	6	6	6	0	0	73	1400	1567
Gemini 1500 M	3	3	3	3	3	3	5	2	3	5	5	5	3	5	0	6	6	3	6	0	0	72	1500	1873
Gemini 1600 M	3	3	3	3	3	5	5	2	4	5	5	5	3	5	0	3	6	3	6	0	0	72	1600	1873
Gemini 1700 M	3	3	3	3	3	3	5	5	3	5	5	5	3	5	3	6	0	3	6	0	0	72	1700	2080
Gemini 1800 M	3	3	3	3	3	5	1	2	5	5	5	5	3	4	0	6	6	3	6	0	0	71	1800	2516
Copilot 1400 F	3	3	3	3	3	5	5	3	3	4	5	5	3	5	3	3	0	6	6	0	0	71	1400	1257
Copilot 1500 F	3	3	3	3	3	5	5	5	2	5	5	5	2	5	3	3	0	6	6	0	0	72	1500	1470
Copilot 1600 F	3	3	3	3	3	5	5	3	4	3	5	5	3	5	3	3	0	6	6	0	0	71	1600	1320
Copilot 1700 F	3	3	3	3	3	5	5	5	4	5	5	5	3	5	0	3	0	6	6	0	0	72	1700	1568
Copilot 1800 F	3	3	3	3	3	5	5	5	4	5	5	5	3	5	0	3	0	6	6	0	0	72	1800	1831
Copilot 1400 M	3	3	3	3	3	5	5	3	2	4	5	5	2	5	3	3	0	6	6	0	0	69	1400	1160
Copilot 1500 M	3	3	3	3	3	5	5	3	3	5	5	5	3	5	0	3	0	6	6	0	0	69	1500	1368
Copilot 1600 M	3	3	3	3	3	5	5	3	3	4	5	5	3	5	0	3	0	6	6	0	0	68	1600	1391
Copilot 1700 M	3	3	3	3	3	5	5	5	4	5	5	5	2	5	0	3	6	6	6	0	2	79	1700	1635
Copilot 1800 M	3	3	3	3	3	5	5	5	4	4	5	5	2	5	0	3	6	6	6	0	2	78	1800	1631
Chatgpt 1400 F	3	3	3	3	3	5	5	3	2	5	5	5	3	5	0	3	6	6	3	0	0	71	1400	1386
Chatgpt 1500 F	3	3	3	3	3	5	5	3	2	5	5	5	3	5	0	3	3	6	3	0	0	68	1500	1408
Chatgpt 1600 F	3	3	3	3	3	5	5	5	3	5	5	5	4	5	0	6	6	6	3	0	0	78	1600	1588
Chatgpt 1700 F	3	3	3	3	3	5	5	5	3	5	5	5	2	5	0	3	6	6	3	0	0	73	1700	1542
Chatgpt 1800 F	3	3	3	3	3	5	5	5	4	5	5	5	3	5	0	3	0	6	3	0	0	69	1800	1667
Chatgpt 1400 M	3	3	0	3	3	5	5	5	0	4	5	5	2	5	0	3	0	6	6	0	0	63	1400	1168
Chatgpt 1500 M	3	3	3	3	3	5	5	5	2	3	5	5	3	5	0	3	0	6	6	0	0	68	1500	1340
Chatgpt 1600 M	3	3	3	3	3	5	5	5	3	5	5	5	3	5	0	3	0	6	6	0	0	71	1600	1481
Chatgpt 1700 M	3	3	3	3	3	5	5	5	3	4	5	5	3	5	0	3	0	6	6	0	0	70	1700	1586
Chatgpt 1800 M	3	3	3	3	3	5	5	5	5	4	5	5	3	5	0	6	6	6	6	0	0	81	1800	1599
